# Application of Moyer’s mixed dentition analysis and establishing probability tables in a sample of Pakistani population

**DOI:** 10.12669/pjms.39.5.6380

**Published:** 2023

**Authors:** Syeda Areeba Rehan, Roofia Imtiaz, Sabeen Mustafa, Aimal Saleh

**Affiliations:** 1 Dr. Syeda Areeba Rehan, BDS. Army Medical College, National University of Medical Sciences (NUMS), Rawalpindi, Pakistan; 2 Dr. Roofia Imtiaz, BDS. Army Medical College, National University of Medical Sciences (NUMS), Rawalpindi, Pakistan; 3 Dr. Sabeen Mustafa, BDS. Army Medical College, National University of Medical Sciences (NUMS), Rawalpindi, Pakistan; 4 Dr. Aimal Saleh, BDS. Army Medical College, National University of Medical Sciences (NUMS), Rawalpindi, Pakistan

**Keywords:** Mixed Dentition, Linear Regressions, Odontometry, Space Maintenance, Space Closure

## Abstract

**Objective:**

To assess the congruity of Moyers’ dental analysis in Pakistani individuals and to make tables foreseeing the size of non-erupted permanent premolars and canines in children.

**Methods:**

A cross-sectional study was conducted at Orthodontics Department of Armed Forces Institute of Dentistry (AFID) Rawalpindi, Pakistan. This was conducted from January 2020 to December 2021 and included mixed dentition subjects’ casts. Mesiodistal widths (MDW) of the permanent dentition were estimated and contrasted with the anticipated values that emerged from the Moyer’s mixed dentition examination. Students t-test, simple linear regression and Pearson correlation coefficient examination were utilized for statistical correlation. All data was gathered utilizing an electronic digital caliper and further investigated through IBM SPSS Statistics 24.

**Results:**

Dental casts of the 258 subjects, 106 (41%) males and 152 (58.9%) females with the ages ranging 15.825±2.95 years were analyzed. Tooth size contrasts were significant (p <0.05) which was noticed in comparison to Moyer’s predictive table. This demonstrates a bigger tooth size in young men than in young ladies. New probability tables were created through linear regression equations for our population.

**Conclusion:**

Moyer’s prediction cannot be applied to the Pakistani population. Mandibular incisors in addition to maxillary first molars are superior in prediction for the size assessment of canines and premolars (cp) that are unerupted in males and upper arch only in females. Another noteworthy conclusion is that in our study sample, in lower arch of females, the width of mandibular molars with mandibular incisors predicts better.

## INTRODUCTION

A mixed dentition stage is known as the time when both deciduous and permanent dentitions exist in the mouth (most often between six to twelve years of age).^1^ Any hereditary or procured inconsistency during this time can be identified in the mixed or permanent dentition. Examination of the mixed dentition decides the space insufficiency or overcrowding that may be occur in the permanent dentition. Henceforth it is an important guide in interceptive orthodontic treatment planning which comprises of sequential extractions, space supervision and space regaining.^1^

In the past, different kinds of analyses have been employed to compute space inconsistency between mixed dentitions. Using a few strategies (radiographic techniques, Tanaka Johnston method, Moyer’s prediction tables) we can figure the MDW of permanent canine and premolars (cp) that have not erupted yet. The most accredited method is the Moyers Mixed Dentition Analysis.^1^-^3^

These investigations have their benefits and drawbacks. Among these, Moyer’s prediction tables are extremely appropriate to utilize. However, it is noteworthy that the conditions have been derived from a Caucasian populace.^1^,^2^ Thus, they are not relevant to others regions, specifically in Pakistan.^4^,^5^

We are aware that the sizes of a tooth contrast significantly between different ethnic communities all over the world. Added to this, recent research shows the diameters of mandibular incisors are poor indicators of MDW of unerupted permanent premolars and canines.^6^ Certain former investigations that were directed on a Pakistani Population have concluded that these analysis do not exactly predict the widths of unerupted cp in our sample.^4^ Therefore, the aim of our research was to mediate the appropriateness of Moyers analysis on Pakistani populace and to form probability tables established from very pertinent regression equations with teeth that precisely anticipate the size of succedaneous teeth for our population.^2^

## METHODS

This cross-sectional study was conducted at a tertiary care hospital, Armed Forces Institute of Dentistry in Rawalpindi, Pakistan. It was a cross-sectional study and commenced from January 2020 till December 2021.

### Ethical approval

After being approved by the Institutional ethical committee (IRB form no. 918/Trg-ABP 1K2), the sample size was calculated using IBM SPSS Statistics 24 based on 95% confidence interval and 0.10 absolute precision. A total of 258 dental casts of patients were obtained through random sampling from the department of orthodontics. These included participants with mixed dentition (152 females and 106 males). The average age of participants was 15.82±2.95 years.

### Inclusion criteria:

- Completely erupted maxillary and mandibular molars, premolars, lower incisors, and canines
- Angle’s class (I, II and III canine and molar relationships)

### Exclusion criteria:

- Malformed, decayed, broken or chipped teeth
- Congenital abnormality or missing teeth
- Clefts
- Teeth with previous restoration
- Former orthodontic treatment

The MD widths of central and lateral incisors (mandibular), canines, premolars alongside first molars were independently estimated from both upper and lower casts. Human error was lessened by using a computerized caliper (Digilog Electronics) for all estimations. It was adjusted to the closest 0.02mm. The estimations were made as per Jensen et al.^7^ To obtain a single value for mandibular and maxillary canine, premolars,1^st^ molar and mandibular incisors, an average of the acquired values was calculated.

To maintain interobserver reliability, 20 sets of study casts were arbitrarily chosen from the 258 sample, measured thrice by three separate observers with a break of one day between each study cast. The interobserver predisposition was determined with the Cohen’s Kappa statistical analysis where the value of 1.00 displays excellent agreement.

All collected data was analyzed through IBM SPSS Statistics 24. Kolmogorov-Smirnov test was done to evaluate normality of data for all dependent and independent variables in the two genders. Teeth were separated into MDW scales as dependent and independent variables. The sum of MDW of premolars and canines each in the mandible (Lcpw) and maxilla (Ucpw) were dependent variables. The sum of first molar (Lim) and mandibular incisors and (Li), the maxillary first molar (LiUm) and mandibular incisors were independent variables.

Information for MDW was expressed through mean ± S.D. To assess the distinction in tooth size across gender, an independent t-test was run. A paired t-test determined the change in tooth size on both sides between the two sexes.

To identify the relationship between the variables in both genders, a Pearson correlation coefficient was used. With previously mentioned variables, a simple linear regression analysis was run.^8^,^9^ The group that had the highest R square value and the least margin of error was considered in both sexes independently. P-value ≤ 0.05 and 95% confidence intervals were significant. For each gender and arch, regression equations were developed separately.^10^ The following was defined as a regression equation:

**Y= a+bx**

Were,

Y----Dependent variable

a----Constant

b----Regression coefficient

x----Independent variable

A table was designed to represent the proposed mixed dentition examination for the Pakistani populace.

## RESULTS

The sample of the current research included 258 dental casts with 106 males (41%) and 152 females (59%). Their average ages ranged from 15.9±3.0 (males) and 15.7±2.7 (females) years. A significant tooth size distinction was detected between sexes. Males displayed bigger first molar and canines in mandible and left canine in upper arch. This was in contrast to that found in females at the degree of separate tooth size (p value<0.05) (Table-I), and the joined width of teeth. There was also a notable variance in both genders regarding the mesiodistal diameters contralaterally in dental arch (p value<0.05) in for certain teeth. The average of each sort of tooth was obtained for simple linear regression analysis.

**Table-I:** Comparison of teeth sizes between boys and girls

Arch	Teeth (FDI system)	Male		Female		P-value

		Mean	S.D	Mean	S.D	
Maxilla	16	10.31	0.66	10.11	0.67	0.018*
	26	10.29	0.77	10.09	0.79	0.050*
	15	6.54	0.73	6.58	0.68	0.669
	25	6.62	0.54	6.56	0.70	0.466
	14	6.90	0.66	6.90	0.58	0.952
	24	7.02	0.59	6.90	0.60	0.133
	13	7.76	0.67	7.62	0.62	0.094
	14	7.75	0.69	7.49	0.58	0.001*
	46	10.65	0.73	10.53	0.63	0.161
	36	10.56	0.80	10.54	0.71	0.781
	45	6.86	0.63	6.75	0.69	0.204
	35	7.01	0.78	6.88	0.81	0.190
	44	7.07	0.62	6.98	0.56	0.257
	34	7.06	0.58	6.94	0.71	0.136
Mandible	43	6.89	0.63	6.66	0.60	0.003*
	33	6.90	0.53	6.66	0.65	0.002*
	42	5.94	0.59	5.91	0.69	0.729
	32	5.98	0.74	5.78	0.69	0.026*
	41	5.59	0.77	5.53	0.90	0.533
	31	5.64	0.85	5.44	0.87	0.082

* Correlation is significant at the 0.05 level.

Mandibular and maxillary cp widths were regressed on three indicators (Table-II). Pearson correlation coefficient demonstrated LiUm to be the prime predictor (p<0.05) of the widths of premolars and canines (Lcpw/Ucpw) among other independent variables (Li, Lim, LiUm).

**Table-II:** Using Pearson Coefficient to correlate Dependent and Independent variables.

Independent variable		Males (N=106)		Females (N=152)		Whole Sample (N= 258)	

		Ucpw	Lcpw	Ucpw	Lcpw	Ucpw	Lcpw
**Li**	Pearson Correlation	0.145	0.360**	0.175*	0.539**	0.171**	0.482**
	p value	0.069	0.000	0.031	0.000	0.003	0.000
**Lim**	Pearson Correlation	0.262**	0.430**	0.196*	0.583**	0.230**	0.532**
	p value	0.003	0.000	0.015	0.000	0.000	0.000
**LiUm**	Pearson Correlation	0.271**	0.447**	0.258**	0.553**	0.271**	0.525**
	p value	0.002	0.000	0.001	0.000	0.000	0.000

* Correlation is significant at the 0.05 level.

** Correlation is significant at the 0.01 level.

Regression models were developed for identifying the diameters of upper (Ucpw) and lower (Lcpw) canines and premolars (dependent factors) in the two sexes. The independent variables belonged to 1 (Li), 2 (Lim) and 3 (LiUm). For the dependent variables in both genders, model 3 (LiUm) had the highest R2 and lowest standard error. Model 2 (Lim) in females was an exemption which firmly anticipated Lcpw than other groups. Simple regression equation was produced for the assessment of Lcpw and Ucpw for both males and females independently relating to distinction in gender and tooth size as shown in Table-III.

**Table-III:** Linear Equation Models.

Maxillary Canine & Premolar (Ucpw)							

BOYS				GIRLS			

Models	SEE	R^2^	Regression Equations	SEE		R^2^	Regression Equations
**Model A**	1.370	0.021	Ucpw= 19.311+ 0.086Li	1.339		0.031	Ucpw = 19.051+0.088 Li
**Model B**	1.377	0.068	Ucpw= 15.963+ 0.241Lim	1.334		0.038	Ucpw =17.365+0.168 Lim
**Model C**	1.333	0.073	Ucpw= 15.677+ 0.257LiUm	1.314		0.067	Ucpw =16.580+0.208 LiUm

**Mandibular Canine & Premolar (Lcpw)**							

**BOYS**				**GIRLS**			

**Models**	**SEE**	**R^2^**	**Regression Equations**	**SEE**	**R^2^**		**Regression Equations**
**Model A**	1.257	0.129	Lcpw= 16.098+ 0.208Li	1.225	0.290		Lcpw =13.894+0.289 Li
**Model B**	1.216	0.185	Lcpw= 12.356+ 0.386Lim	1.182	0.340		Lcpw = 8.764+ 0.534 Lim
**Model C**	1.205	0.200	Lcpw= 11.896+ 0.413LiUm	1.212	0.305		Lcpw =10.220+0.477 LiUm

***Regression equations derived as:***

***Upper dentition:*** Females: Y = 16.580+0.208x, Males: Y = 15.677+ 0.257x

***Lower dentition:*** Females: Y = 8.764+ 0.534x, Males: Y = 11.869+ 0.413x.

Using the above linear equations, new probability tables were made according to Moyer’s template (Table-IV).

**Table-IV:** New Probability tables established for Pakistani population.

Sum of LiUm in mm (except Lim in mandible girls)	Male		Female	

	Ucpw	Lcpw	Ucpw	Lcpw
19	20.56	19.71	20.53	18.91
19.5	20.68	19.92	20.63	19.17
20	20.81	20.12	20.74	19.40
20.5	20.94	20.33	20.84	19.71
21	21.07	20.54	20.94	19.97
21.5	21.20	20.74	21.05	20.24
22	21.33	20.95	21.15	20.51
22.5	21.45	21.16	21.26	20.77
23	21.58	21.36	21.36	21.04
23.5	21.71	21.57	21.46	21.31
24	21.84	21.78	21.57	21.58
24.5	21.97	21.98	21.67	21.84
25	22.10	22.19	21.78	22.11
25.5	22.23	22.40	21.88	22.38
26	22.35	22.60	21.98	22.64
26.5	22.48	22.80	22.09	22.91
27	22.61	23.02	22.19	23.18
27.5	22.74	23.22	22.30	23.44
28	22.87	23.43	22.40	23.71
28.5	23.00	23.63	22.50	23.98
29	23.13	23.84	22.61	24.25

## DISCUSSION

Moyer’s analysis of un-erupted permanent dentition in the early mixed dentition are the most widely employed of the numerous mixed-dentition analysis documented in many studies.^2^,^6^,^9^,^11^ Preliminary literature has proven the analysis in-applicability in a Pakistani population.^4^ As a result, our work was aimed at applying their principles to a Pakistani sample and generating new prediction tables appropriate for our community.

The MDWs of permanent teeth differ between groups based on race and ethnicity.^6^,^8^,^9^,^11^,^12^ When compared to demographic groups from India, Indonesia, Saudi Arabia, Syria, Taiwan and Nepal, our sample had smaller mean values for the mesiodistal lengths of canines, premolars, and mandibular incisors.^4^,^8^,^11^-^13^ One study from Pakistan’s Sindh province did not identify any significant variations in tooth size between genders.^5^ However, our research in the Punjab region found that males had wider MDWs than females. Because the present study’s findings are in line with those of previous researches that have found differences between the sexes, data was processed and linear equations were constructed separately for gender in the current study.

Numerous researchers suggested measuring the mesiodistal breadth of teeth on dental models with electronic digital calipers to increase accuracy.^14^,^15^ As other researchers have noted, the difference between tooth size in the right and left sides in the current study was very small and statistically insignificant.^8^,^10^ As a result, the calculation used the mean value of the MD diameters of these teeth.

Orthodontic literature has made use of various tooth arrangements. Using MDWs of lower incisors as a basis in the American population, Moyers and Tanaka & Johnston created a regression table and equation.^1^-^3^ Numerous researchers have generated linear regression models for their particular populations based on four Li because they questioned that whether these prediction equations are applicable to cultural and racial disparities in tooth size. Lower incisors are not a better predictor for the estimation of the MDW of un-erupted canines and premolars, according to a recent report from several researchers.^8^-^11^ These researchers introduced additional predictors with a higher degree of accuracy, namely the mandibular and maxillary permanent first molars.^10^ To determine which tooth combinations in Pakistani populations provide the best predictions, various tooth combinations were used as predictors in the current study, and values of r, R2, and SEE were recorded. We observed that regression equation centered on the width of maxillary first molars more precisely anticipate the size of both Ucps, Lcps in boys and Ucps in young female adolescents. However, regression equation based on the width of mandibular first molar and incisors precisely predict the size of Lcps in young females.

The findings of current study concur with those of other studies that claimed that only the mandibular permanent incisors are not better predictors of outcome.^8^,^10^,^16^ According to other radiology and non-radio-graphic methods, the difference between the actual and predicted value in the current study, which used the maxillary first molar as predictors, is among the smallest overall, except in the mandible in girls, where mandibular incisors and the maxillary first molar were the best predictors. In comparison to mandibular first molars, maxillary first molars have an advantage because mandibular first molars are harder to measure because they are hidden by the distal gingival groove and are more susceptible to dental caries. Therefore, maxillary first molars are a better alternative predictor with a higher correlation coefficient.^10^

Despite the ethnic diversity in our sample, these prediction tables, which are based on data from a Pakistani sample, should be reliable when used in local children. The probability table is simple to understand and apply without need for equation memorization.^17^ In order to get more appropriate tooth size statistics for Pakistani population, additional studies with bigger samples, representing more ethnic subgroups, are required. Clinicians in Pakistan should exercise caution when using these linear regression equations. It should be determined whether the patient satisfies the requirements for becoming one of our subjects. We advise conducting validation studies (based on comparable samples) to verify the suitability and accuracy of the modified linear regression models. In order to make these equations more applicable to a wider range of ethnic groups in Pakistan, it is also necessary to test their accuracy as well.

### Limitations

It is noteworthy that this study is not to be considered as a guide for another ethnicity as it was based on a small sample. It is proposed that more studies be conducted to investigate a larger population.

## CONCLUSION

The prediction charts of Moyers in a sample of Pakistani adolescents, was unable to predict the MDWs of unerupted canines and premolars. New probability tables must be proposed which are appliable to the Pakistani population. The prediction equation based on maxillary first molar and mandibular incisors precisely predicts un-erupted premolar and canine width in both arches in boys and only in maxilla in girls. An equation centered on mandibular first molar and mandibular incisors predict lower premolar and canine width better in girls compared with lower incisors alone.

### Authors’ Contribution:

**SA** is primarily responsible for the construction of this research, data collection, statistical analysis and formation of tables.

**RI, SM and AS** worked in data collection and drafting of manuscript.

All authors are involved in final approval of manuscript.

## References

[1] Arangannal P (2019). Mixed Dentition Analysis Procedure:A Review. Indian J Public Health Res Dev.

[2] Moyers RE (1988). Handbook of orthodontics.

[3] Tanaka MM, Johnston LE (1974). The prediction of the size of unerupted canines and premolars in a contemporary orthodontic population. J Am Dent Assoc.

[4] Bano S, Ahmed A, Ali F, Afzal S, Rasool G (2021). Applicability of Moyers'prediction table in orthodontic patients reporting to KCD Peshawar population. J Rehman Coll Dent.

[5] Mahida H, Memon S, Khan M, Naz F (2021). Applicability of two non-radiographic mixed dentition analysis methods in orthodontic patients. Pak J Med Dent.

[6] Ravinthar K, Gurunathan D (2020). Applicability of different mixed dentition analyses among children aged 11–13 years in Chennai population. Int J Clin Pediatr Dent.

[7] Moorrees CF, Thomsen SØ, Jensen E, Yen PKJ (1957). Mesiodistal crown diameters of the deciduous and permanent teeth in individuals. Journal of Dental Research.

[8] Chong SY, Aung LM, Pan Y-H, Chang W-J, Tsai C-Y (2021). Equation for tooth size prediction from mixed dentition analysis for Taiwanese population:A pilot study. Int J Environ Res Public Health.

[9] Hashim HA, Al-Hussain HA-N, Hashim MHA (2019). Prediction of the size of unerupted permanent canines and premolars in a Qatari sample. Int J Orthod Rehabil.

[10] Shetty RM, Daga P, Reddy H, Yadadi SS, Lakade L, Shetty SR (2019). A newly proposed regression equation for mixed dentition analysis using the sum of the width of permanent mandibular central incisors and permanent mandibular first molars as a predictor of width of unerupted canine and premolars. Pesqui Bras Odontopediatria Clin Integr.

[11] Handayani FT, Hidayah RA (2019). Applicability of Moyers and Tanaka-Johnston analyses for the Arab population of Pekalongan, Indonesia. Dent J (Majalah Kedokteran Gigi).

[12] Doda A, Saraf BG, Indushekhar K, Sheoran N, Sardana D, Kumar T (2021). Evaluation and Applicability of Tanaka–Johnston and Moyers'Mixed Dentition Analysis for North Indian Population. World.

[13] Al-Dlaigan YH, Alqahtani ND, Almoammar K, Al-Jewair T, Salamah FB, Alswilem M (2015). Validity of moyers mixed dentition analysis for Saudi population. Pak J Med Sci.

[14] Dhillon SK, Kahlon SS, Sharma K, Boparai CDS, Kaur D (2022). Assessment of accuracy and reliabilty of measurements obtained on 3D scanned models to conventional models. Int J Health Sci.

[15] Liu J, Liu Y, Wang J, Zuo X, Wang X, Zhang Y (2021). Dental measurements based on a three-dimensional digital technique:A comparative study on reliability and validity. Arch Oral Biol.

[16] Butt S, Javed M, Ijaz S, Awais F, Wahid A, Aziz S, Khan AA (2016). Mixed Dentition Space Analysis in Adolescents of Lahore, Pakistan. Saudi J Oral Dent Res.

[17] Bherwani AK, Fida M (2011). Development of a prediction equation for the mixed dentition in a Pakistani sample. Am J Orthod Dentofacial Orthop.

